# Isolation and Characterization of Bacteria Colonizing *Acartia tonsa* Copepod Eggs and Displaying Antagonist Effects against *Vibrio anguillarum*, *Vibrio alginolyticus* and Other Pathogenic Strains

**DOI:** 10.3389/fmicb.2017.01919

**Published:** 2017-10-06

**Authors:** Mahammed Zidour, Mickaël Chevalier, Yanath Belguesmia, Benoit Cudennec, Thierry Grard, Djamel Drider, Sami Souissi, Christophe Flahaut

**Affiliations:** ^1^Université d’Artois, INRA, ISA, Université Lille 1, Université du Littoral Côte d’Opale, EA 7394 – ICV – Institut Charles Viollette, Lille, France; ^2^Université Lille 1, CNRS, Université du Littoral Côte d’Opale, Laboratoire d’Océanologie et de Géosciences, UMR 8187 LOG, Wimereux, France

**Keywords:** *Acartia tonsa*, copepod eggs, MALDI-TOF-MS, *Bacillus pumilus*, antagonism, antibacterial compounds, amicoumacin

## Abstract

Copepods represent a major source of food for many aquatic species of commercial interest for aquaculture such as mysis shrimp and early stages of fishes. For the purpose of this study, the culturable mesophilic bacterial flora colonizing *Acartia tonsa* copepod eggs was isolated and identified. A total of 175 isolates were characterized based on their morphological and biochemical traits. The majority of these isolates (70%) were Gram-negative bacteria. Matrix-assisted laser desorption/ionization-time of flight-mass spectrometry (MALDI-TOF-MS) was used for rapid identification of bacterial isolates. Here, 58% of isolates were successfully identified at the genus level and among them, 54% were identified at the species level. These isolates belong to 12 different genera and 29 species. Five strains, identified as *Bacillus pumilus*, named 18 COPS, 35A COPS, 35R COPS, 38 COPS, and 40A COPS, showed strong antagonisms against several potential fish pathogens including *Vibrio alginolyticus, V. anguillarum*, *Listeria monocytogenes*, and *Staphylococcus aureus*. Furthermore, using a differential approach, we show that the antimicrobial activity of the 35R COPS strain is linked primarily to the production of antimicrobial compounds of the amicoumacin family, as demonstrated by the specific UV-absorbance and the MS/MS fragmentation patterns of these compounds.

## Introduction

In aquatic ecosystems, basal trophic levels are constituted by microscopic organisms called plankton which consist of a plant part, the phytoplankton, represented by unicellular algae and an animal part, the zooplankton. This latter is the main aquatic compartment in terms of biomass and diversity (Mauchline, 1998) especially for the crustaceans subphylum where the copepod subclass represents the majority of these organisms (Humes, 1994). Copepods could be found in a variety of habitats and are adapted to specific types such as fresh or deep water. They have different feeding regimes and can live freely in the water column or in the sediment (Mauchline, 1998) but can also be parasites, such as sea lice, of aquatic organisms (González et al., 2016). Copepods play an important role in the marine ecosystem given its middle trophic position between primary producers (phytoplankton), microbial food webs and higher trophic levels (Roddie et al., 1984). Copepods are also the focus of many ecological studies because of their key position in marine food webs, being important grazers as well as a food source for higher trophic levels (Souissi et al., 2016).

The order of calanoid copepods is largely abundant in marine and brackish environments and therefore plays a major role in aquatic food webs but also in the diet of many commercial fish species which are dependent on calanoid copepods in either their larval or adult forms. The calanoid copepod *Acartia tonsa* is recognized as an emerging biological model, a source of live prey for aquaculture purposes (Drillet et al., 2006) and is widely used for evaluation of marine contaminants (Stancheva et al., 2015). They have a good biochemical composition in terms of essential fatty acids. These characteristics make the microflora of these organisms of prime interest to evaluate infection risks and transmission by food intake.

Aquaculture is beset by infectious diseases which are a major impediment to its development and are one of the most significant causes of economic losses. These infectious diseases are caused by different bacterial and viral pathogens. *Vibrio* species especially *V. harveyi, V. alginolyticus*, and *V. parahaemolyticus* are well characterized as major causes of bacterial infections in fish farms (Carbone and Faggio, 2016). Interestingly, several *Vibrio* species, including pathogenic ones, are also commonly found in a variety of copepods (Colwell and Huq, 1998; Gugliandolo et al., 2008) including *A. tonsa* which hosts various other bacterial genera like *Pseudomonas* and *Flavobacterium* (Roddie et al., 1984). Nonetheless, the microflora of the copepods is poorly documented and the few studies focused on the adult state (Skovgaard et al., 2015). Calanoid copepods from the genus *Acartia* are well studied because they are promising live feeds in marine larviculture (Hansen et al., 2016). Recently, the use of quiescent eggs of copepods and mainly *A. tonsa* is suggested as promising technology to be used in hatcheries due to their immediate response to stimulation signals of induction treatments and rapid hatching after the quiescent phase (Drillet et al., 2006; Højgaard et al., 2008; Abate et al., 2015). In fact, we did not find in the literature any study focusing on the pathway of bacteria colonization in copepod eggs. But, some recent studies focusing on late developmental stages of copepods (mainly adults) showed that the colonization pathway could be complicated (Rawlings et al., 2007; Almada and Tarrant, 2016). Moreover, some experimental studies confirmed that some bacterial strains (such as *Escherichia coli*.) cannot colonize copepods (Dumontet et al., 1996). However, the pathway of bacterial colonization of copepod eggs was not the central element of our study. In fact, we targeted the eggs of the calanoid species *A. tonsa* as the high potential of using these eggs mostly storage in cold (2–4°C) as quiescent eggs, in aquaculture use is intensively studied. Especially the effect of stocking density on production of *A. tonsa* eggs was studied and the effect of cold storage duration on the hatching success of eggs was reviewed in (Hansen et al., 2016). Other studies focused on the effect of external conditions (elevated temperature, wavelength, light intensity etc.) on the hatching success of freshly produced or cold-stored eggs of *A. tonsa* (Hagemann et al., 2016; Hansen et al., 2016; Franco et al., 2017). In order to achieve the great potential of *A. tonsa* eggs in aquaculture, their microbiome should be studied for two main reasons: (i) in order to screen the presence of pathogens that may introduce any risk in hatchery if no disinfection was used and (ii) to look if some associated strains have positive effect (like probiotic potential) and consequently they have to be maintained with eggs. In fact, some studies already mentioned that encapsulation of probiotic bacteria of genus *Bacillus* through copepods is very efficient in improving survival of fish larvae such as grouper (Sun et al., 2013). However, to our knowledge the isolation of bacteria strains from copepod eggs (or copepods in general) with high probiotic potential has never been done in the past.

As in other areas of animal farming, antibiotics have routinely been administered but there have negative impacts on the environment and human health. This includes the emergence of antibiotic-resistant bacterial strains, the accumulation of residues in edible tissues and the depression of immune systems (Carbone and Faggio, 2016). For these reasons, the use of antibiotic treatment in aquaculture was banished in the European Union and is under stringent regulations in the United States of America and in other countries (Carbone and Faggio, 2016). This strict regulation led to the investigation and the development of alternative strategies to disease control. Among these alternative control regimes, vaccination and the use of anti-microbial agents were initially proposed (Austin and Austin, 1993). Probiotic bacteria also appear as an interesting strategy as they can be a natural protective and/or curative treatment, based on their beneficial properties. Probiotics used in aquaculture belong to a number of phylogenetic lineages, but mainly derive from two bacterial divisions: the proteobacteria (especially gammaproteobacteria) and the Firmicutes, among which numerous members of the genus *Bacillus* have been described (Hong et al., 2005) such as *Bacillus subtilis* (Moriarty, 1998; Balcázar and Rojas-Luna, 2007; Aly et al., 2008) and *B. pumilus* (Duc et al., 2004). Although probiotic propensity is not solely related to molecules produced by a strain, *B. pumilus* species produce structurally diverse classes of secondary metabolites, such as fengycins, surfactants, glycocholic acid, amicoumacins A and B and lipoamides A–C and these structurally polyvalent compounds have effective antimicrobial activity against various pathogens (Lebbadi et al., 1994; Berrue et al., 2009; Xu et al., 2014; Mora et al., 2015).

The aim of the present study was to isolate and characterize the culturable mesophilic bacterial flora colonizing *A. tonsa* eggs issued from the copepod species collection of the LOG-Marine Station of Wimereux located on northern coast of France. To fulfill this goal, bacterial isolation was performed using nutritive agar medium at 25°C for 48 to 72 h. Gram staining and catalase test were performed prior to further characterization of the isolates. MALDI-TOF-MS was used as main means of bacterial identification, along with some 16S rDNA molecular identification. The antagonism of strains was tested against selected fish pathogens and molecules responsible for antagonisms were characterized by liquid chromatography coupled to mass spectrometry.

## Materials and Methods

### Conditions of Copepod Breeding and Copepod Egg Collection

The *A. tonsa* strain used in the present study was obtained from Roskilde University (strain code DFH-ATI) and was raised at the marine station of Wimereux for several generations. Copepods were routinely cultivated in 20-L polycarbonate carboys and then upscaled up to 300-L volume by using transparent tanks as described by (Tlili et al., 2016) excepted that copepods were fed every 2 days with an excess of the alga *Rhodomonas* sp. *A. tonsa* eggs were collected by sieving the culture water using the same procedure as in Pan et al. (2015). Then eggs were rinsed using sterile sea water and stored at 4°C in dark sterile bottles of 15 mL.

### Growth Conditions and *Bacillus* Isolation

Copepod eggs were washed three times with sterile distilled water and then mixed for 3 min using a homogenizer Ultra-turrax Ika-T25D model (Imlab, Lille, France) in 9 mL of SSB (1 g tryptone, 8.5 g sodium chloride dissolved in 1 L of distilled water), serially diluted in 0.9% (v/v) SSB from 10^-1^ to 10^-6^, plated on nutrient agar in Petri dishes (Sigma–Aldrich, St. Quentin Fallavier, France) using the spread plate method and incubated at 25°C for 48 h. Morphological characteristics of isolates were determined using visual assessment of bacterial colonies on nutrient agar plates along with microscopic examination according to the standard procedures described in Bergey’s manual of systematic bacteriology. Gram staining and the catalase test (catalase production) were carried out. Morphologically different colonies were selected from the agar plates, streaked on fresh nutrient agar plates and incubated at 25°C for 48 h. Finally, bacterial isolates were inoculated into nutrient broth (Merck, Germany) and stored in 30% glycerol at -80°C for further analysis.

### Bacteria Identification by MALDI-TOF-MS

#### Mass Spectrometry (MS) Profile Acquisition from Colony Forming Units

Single bacterial colonies were picked up three times with pipette tips and separately smeared as a thin layer onto a ground steel matrix-assisted-laser-desorption/ionization (MALDI) target according to the manufacturer’s instructions (Bruker Daltonics, Bremen, Germany). The on-target deposits were overlaid with 1 μL of 70% formic acid solution, dried at room temperature, overlaid again with 1 μL of matrix solution [10 mg/ml of HCCA dissolved in ACN/water/TFA (50/47.5/2.5; v/v/v)] and dried again (Nacef et al., 2016).

Matrix-assisted laser desorption/ionization-time of flight-mass spectrometry analyses were performed on an Autoflex Speed^TM^ (Bruker Daltonics) running Flexcontrol 3.4 software. The MALDI-TOF mass spectrometer calibration was performed using the BTS as per Bruker’s recommendations. This calibration kit contains a typical protein extract of *E. coli* DH5alpha spiked with two additional pure proteins (RNAse A and myoglobin) to cover an overall mass range from 4 to 17 kDa. MALDI-MS profiles were acquired in positive linear mode across the m/z range of 2,000–20,000 Da using the manufacturer’s automatic method MBT_FC.par. Each MALDI-MS profile was the sum of the ions obtained from 480 laser shots performed randomly on different regions of the same spot.

#### Bacteria Identification Using Biotyper

Mass spectra were processed using Biotyper software (version 3.0; Bruker Daltonics) running with the Biotyper database version DB-5989, containing 5989 reference MALDI-MS profiles (5298 of bacteria, 626 of yeasts and 65 of filamentous fungi) called MSP. The experimental MALDI-MS profiles obtained from bacteria isolates were matched on the reference MALDI-MS profiles and the matches were restituted by Biotyper along with a Log(score) and an associated color code (green, yellow, and red). Briefly, a Biotyper Log(score) exceeding 2.3 (green color) indicates a highly probable identification at the species level. A Log(score) between 2.0 and 2.3 corresponds to highly probable identification at the genus level (green color) and probable identification at the species level. A Log(score) between 1.7 and 2.0 (yellow color) implies only probable genus identification; while score value lower than 1.7 (red color) means no significant similarity between the unknown profile and any of those of the database.

### Molecular Biology Experiments

#### Genomic DNA Extraction

Pure genomic DNA was extracted from each *Bacillus* strain identified by MALDI-TOF-MS with the Wizard^®^ Genomic DNA Purification Kit (Promega, Charbonnières-les-Bains, France) and then quantified with a Nanodrop Lite (Biowave II, Biochrom WPA, Cambridge, United Kingdom).

#### Polymerase Chain Reaction (PCR) -Based Amplification of Bacterial 16S Ribosomal DNA (16S rDNA)

The 16S rDNA of the five strains (18 COPS, 35A COPS, 35R COPS, 38 COPS, and 40A COPS) were amplified with 16S forward 5′-AGAGTTTGATCMTGGCTCAG-3′ and 16S reverse 5′-GGMTACCTTGTTACGAYTTC-3′ primers (Drago et al., 2011) using the following PCR program: 94°C/5 min, 29 cycles at 94°C/1 min, 55°C/1 min and 72°C/1 min and finally 72°C/5 min (Al Atya et al., 2015). Amplified products were purified using a PCR purification kit (Qiagen, Courtaboeuf, France) and sequenced by Eurofins Genomics (Munich, Germany). Nucleotide sequences were subjected to homology search using the nucleotide BLAST software of the NCBI. Phylogenetic analysis of 16S rDNA sequences by maximum likelihood analysis method was performed using MEGA 7 according to Hall (Hall, 2013).

### Antagonism Assay

Antibacterial activity was assessed against the GNB and GPB listed in **Table 1**, using two methods: the spot inoculation on agar test and the well-known agar diffusion test slightly modified by Al Atya et al. (2015). The pathogenic bacteria used in this study were handled in a laboratory of microbiological safety level L2 to avoid contamination and to comply with the hygiene and safety of the laboratory according to the safe conditions of use of pathogens.

**Table 1:** Antimicrobial activities of cell free supernatant (CFS) from *Bacillus pumilus* by well agar diffusion method.

Bacteria	*Bacillus pumilus*					*Bacillus subtilis*								
		
Target bacteria	18 COPS	38 COPS	35R COPS	40A COPS	35A COPS	181 COPS	21 COPS	23 COPS	24 COPS	30 COPS	351 COPS	401 COPS	411 COPS	58 COPS
*Vibrio angularum (1 COPS)*	+	++	+	+	+	-	-	-	-	-	-	-	-	-
*Vibrio alginolyticus CIP* 103336	+++	+++	+++	+	+++	-	-	-	-	-	-	-	-	-
*Vibrio alginolyticus (17 EMB)*	+++	+++	+++	+++	+++	-	-	-	-	-	-	-	-	-
*Kocuria rhizophila CIP* 53.45	+++	+++	+++	+++	+++	-	-	-	-	-	-	-	-	-
*Listeria innocua* ATCC 51742	+++	+++	+++	+++	+++	-	-	-	-	-	-	-	-	-
*Listeria monocytogenes* 157	++	+++	++	+++	+++	-	-	-	-	-	-	-	-	-
*Listeria monocytogenes* 158	-	-	-	-	-	-	-	-	-	-	-	-	-	-
*Listeria monocytogenes* 161	-	-	-	-	-	-	-	-	-	-	-	-	-	-
*Listeria monocytogenes* 162	-	-	-	-	-	-	-	-	-	-	-	-	-	-
*Staphylococcus epidermidis*	-	-	-	-	-	-	-	-	-	-	-	-	-	-
*Staphylococcus aureus* ATCC 33862	+	+	++	+	++	-	-	-	-	-	-	-	-	-
*Staphylococcus aureus* ATCC 25923	++	++	++	+	++	-	-	-	-	-	-	-	-	-
*Staphylococcus aureus* (MRSA) S1	-	-	-	-	-	-	-	-	-	-	-	-	-	-
*Staphylococcus aureus* (MRSA) S2	-	-	-	-	-	-	-	-	-	-	-	-	-	-
*Staphylococcus aureus* (MRSA) ATCC 43300	-	-	-	-	-	-	-	-	-	-	-	-	-	-


*(-); no inhibition, (+);4–5 mm inhibition zone, (++); 6–8 mm inhibition zone, (+++); 10–15 mm inhibition zone.*

#### Assessment of Antimicrobial Activity Based on Spot Inoculation on Agar

The antimicrobial activities of *B. pumilus* strains named 18 COPS, 35A COPS, 35R COPS, 38 COPS, and 40A COPS and 9 strains of *B. subtilis* named 181 COPS, 21 COPS, 23 COPS, 24 COPS, 30 COPS, 351 COPS, 401 COPS, 41 COPS, 58 COPS isolated from copepod eggs were determined in triplicates by spot inoculation on agar using 5 μL of *B. pumilus* and *B. subtilis* cultures (collected after 48 h of incubation at 25°C) on Mueller Hinton agar plates pre-inoculated with 150 μL of pathogenic strains listed in **Table 1**. Thereafter, plates were incubated at 25°C for 48 h and the antibacterial activity was determined by measuring the inhibition zone diameters around the spot of *B. pumilus* and *B. subtilis* cultures.

#### Assessment of the Antimicrobial Activity Based on the Agar Diffusion Test

The CFS used for antibacterial activity measurement were obtained by centrifugation (10,000 × *g*, 10 min, 4°C) from fresh cultures of *B. pumilus* and *B. subtilis* grown at 25°C for 24–48 h in nutrient broth. Wells were made within solid Mueller Hinton agar plates, pre-inoculated with 150 μL of pathogenic strains (**Table 1**) and filled with 50 μL of each *B. pumilus* and *B. subtilis* CFS. Agar plates were left at room temperature, in sterile conditions, for 1 h before incubation for 24–48 h at 25°C. Each assay was performed in triplicate. Following incubation, antibacterial activity was assessed by measuring the inhibition zones around the well containing the CFS. The diameter of each zone inhibition was measured in millimeters.

### Characterization of Molecules Responsible for the Antibacterial Activity

The CFS samples from *B. pumilus* and *B. subtilis* cultures were separated by reversed-phase chromatography using an ACQUITY-biocompatible HPLC-system (Waters, Manchester, United Kingdom) equipped with a Phenomenex C18 Kenetex core-shell column (ID 3.0 mm × 150 mm, 2.6 μm, 100 Å). The C18-retained molecule elution was carried out using a linear gradient of ACN containing 0.1% FA (25–95% ACN over 55 min) and a flow rate of 500 μL/min. The HPLC eluent was directly electrosprayed from the column end at an applied voltage of 3 kV, using a desolvation gas (N2) flow of 500 L/h, a nebulier gas flow of 6.5 bar and desolvation temperatures of 150 and 300°C, respectively. The chromatography system was coupled to a SYNAPT-G2-Si mass spectrometer (Waters) operating in data-dependent mode. The full MS survey scans were acquired at a 20,000 (FWHM) resolving power, over the mass range of 300–3,000 m/z. Precursors observed with an intensity over 1,000 counts were selected for ion trap CID fragmentation with an isolation window of 3 amu and subjected to a collision energy ramping from 10 to 20 V for low mass and 40 to 120 V for high mass. The MS/MS spectra were recorded on the 50 to 3000 m/z range. A maximum injection time of 100 ms was used for CID MS^2^ spectra that were acquired over the same mass range. The method was set to analyze the five most intense ions from the survey scan. Peaks were analyzed using Mass Lynx software (ver.4.1; Waters). Structures were drawn using ChemDraw Ultra (Version 12.0.2.1076, Cambridge, MA, United States).

## Results

### Isolates and High-Throughput Identification of Bacteria

#### Bacterial Isolates

Morphological characterization of the *A. tonsa* egg isolates obtained on nutrient agar revealed that, out of a total of 175 strains, 69% are bacilli forms, 19% cocci and 11% coccobacilli. Concomitantly, 30% of isolates are Gram-positive strains and 70% are Gram-negative strains while 27% of the strains appeared to be catalase positive and 73% catalase negative.

#### High-Throughput Identification by MALDI-TOF

**FIGURE 1:**
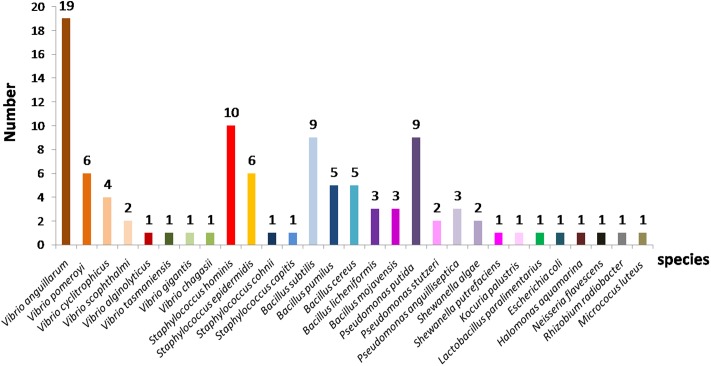
Species distribution of bacterial strains identified by MALDI-TOF mass spectrometry isolated from copepod eggs of *Acartia tonsa*.

Out of the 175 isolates obtained from the copepod eggs, 73 isolates (42%) could not be identified by MALDI-TOF-MS, probably due to the absence of reference mass spectra related to these species in the Bruker database. In contrast, 102 (58%) isolates were identified, of which 54% with certainty at the genus and species levels and 46% only at the genus level. The results of MALDI-TOF-MS-based identifications showed a great diversity of bacterial strains belonging to twelve different genera including Gram-positive and Gram-negative (**Figure 1**), with *Vibrio*, *Staphylococcus* and *Bacillus* as the dominant genera. Amongst the 29 species found, (i) 8 belong to the genus *Vibrio*, notably *V. anguillarum* (19 strains), *V. pomeroyi* (6 strains), *V. cyclitrophicus* (4 strains), *V. alginolyticus* (1 strain), *V. scophthalmi* (2 strains), *V. gigantis* (1 strain) and *V. changasii* (1 strain); (ii) 4 species belong to the genus *Staphylococcus* including *S. hominis* (10 strains), *S. epidermidis* (6 strains), *S. cohnii* (1 strain), and *S. capitis* (1 stain); (iii) 5 species belong to the genus *Bacillus* with the species *B. subtilis* (9 strains), *B. pumilus* (5 strains), *B. licheniformis* (3 strains), *B. cereus* (5 strains), and *B. mojavensis* (3 strains), and (iv) 3 species of the genus *Pseudomonas* such as *P. putida* (9 strains), *P. stutzeri* (2 strains), and *P. anguilliseptica* (3 strains). In addition to these dominant genera, other genera such as *E. coli*, *Shewanella algae, Shewanella putrefaciens*, *Micrococcus luteus*, *Rhizobium radiobacter*, and *Neisseria flavescens* were also unambiguously identified by MALDI-MS profiling.

Some of these bacteria are pathogenic to both fishes and humans (e.g., *Vibrio* spp.), some are harmless to fishes but pathogenic to humans (e.g., *Listeria, Staphylococcus*, and *Vibrio*) while some can be potential probiotics both for fishes and humans (e.g., *Bacillus*). Interestingly, in growing condition close to that of rearing fish (25°C), the dominant genera isolated from the eggs of copepod *A. tonsa* are *Vibrio* and *Bacillus*. Therefore, 14 strains of *Bacillus* genus, including 5 strains of *B. pumilus* named 18 COPS, 35A COPS, 35R COPS, 38 COPS, 40A COPS, and 9 strains of *B. subtilis* named 181 COPS, 21 COPS, 23 COPS, 24 COPS, 30 COPS, 351 COPS, 401 COPS, 41 COPS, and 58 COPS were tested against some fish pathogens to evaluate their antagonistic activities.

### Antagonistic Activities of Isolated *Bacillus* Strains

The antagonistic activities were assessed in triplicates using two methods: the on-site inoculation agar test and the agar diffusion assay, for which the antagonistic activity was quantified using the size of the growth inhibition zone (in mm) around the spot or the well. The spot inoculation agar test revealed that only the *B. pumilus* strains displayed antagonistic activities against *Vibrio*, *Kocuria*, *Listeria*, and *Staphylococcus* (data no shown), demonstrating that the cells and CFS are both responsible for such antagonistic activities. To identify if the antimicrobial activity was due to a secreted molecule, the antimicrobial activity of CFS from *B. pumilus* and *B. subtilis* was assessed by the agar well diffusion method and the results are gathered in the **Table 1**.

As expected, the CFS of *B. subtilis* strains had no activity against our range of pathogens including strains of *Vibrio, Listeria*, and *Staphylococcus*. On the other hand, the CFS of *B. pumilus* strains displayed activities against these pathogens. More precisely, the highest antagonistic activity for the five tested *B. pumilus* strains was observed against *V. alginolyticus* CIP103336 and *V. alginolyticus* 17EMB (previously isolated by ourself from seawater), for which obtained inhibition zones ranged between 10 to 15 mm in size, except for *B. pumilus* 40A COPS which displayed weaker activity against *V. alginolyticus* CIP103336. A similar antagonistic activity pattern was observed against *Listeria innocua* ATCC 51742 and *L. monocytogenes* 157 with wide inhibition zone (10–15 mm). Interestingly, these antagonisms appeared to be strain specific, as no inhibition zone was observed for the other *L. monocytogenes* strains tested (*L. monocytogenes 158, 161, and 162*). The CFS of *B. pumilus* strains displayed activities against *Staphylococcus aureus* ATCC 33862, *S. aureus* ATCC 25923 and *V. anguillarum* 1 COPS (isolated during this study from copepod eggs), for which the sizes of obtained inhibition zones were close to 6–8 mm. These antagonistic activities were lower than observed activities against *V. alginolyticus* strains. No activity was observed for any of the methicillin-resistant *S. aureus* (MRSA) strains tested and the *S. epidermidis* strain.

### Phylogeny of the *B. pumilus* Isolates

**FIGURE 2:**
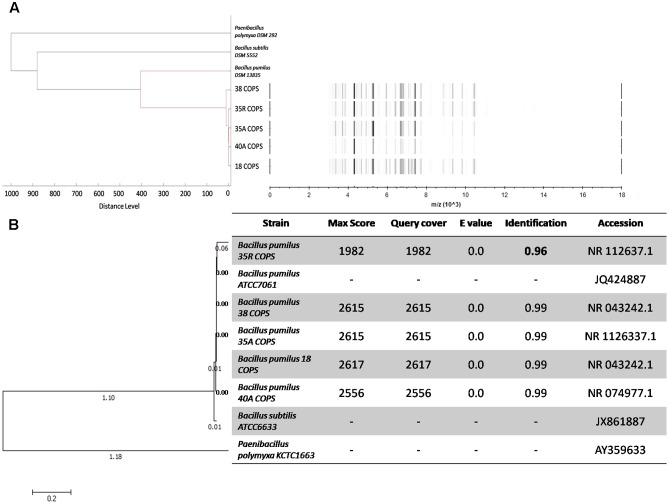
Dendrogram and gel view MALDI-TOF mass spectra comparing the phylogenetic tree of 16S rDNA gene sequences and the phylogeny of *Bacillus pumilus* strains. **(A)** Dendrogram and gel view MALDI-TOF-MS profiles created by the Biotyper MSP Dendrogram Creation Standard Method, version 1.4 with default settings as follow: distance measure: correlation; linkage: –300, related-0. Out-grouping was performed with the reference strain *B. pumilus* DSM1382, *B. subtilis* DSM 5552 and *Paenibacillus polymyxa* DSM 292 **(B)** Neighbor-joining tree and table of alignment scores of 16S rDNA gene sequences of *B. pumilus* strains isolated from copepod eggs (*A. tonsa*) using the nucleotide (BLAST) software of NCBI tree generated using MEGA 7 software. Out-grouping was performed with the reference strain *B. pumilus* ATCC7061, *B. subtilis* ATCC6633 and *Paenibacillus polymyxa* KCTC *1663*.

The *B. pumilus* isolates identified in this study exhibit high activities against pathogenic bacteria both for humans and fishes and especially at larval stages. The gel view (**Figure 2A**, right panel) of MALDI-TOF-MS profiles of *B. pumilus* strains shows that the MS profiles are similar in term of intensity and mass over charge ratio (m/z) of mass signals. Moreover, as demonstrated by the dendrogram displayed in **Figure 2A** (left panel), these *B. pumilus* MS profiles appear highly similar to the Bruker database reference MS profile for *B. pumilus* DSM 13835. Additionally, this dendrogram illustrates, based on the strains MS profiles, the phylogenic distance between two other strains: *Paenibacillus polymyxa* DSM 292 and *B. subtilis* DSM 5552.

To confirm these results, all *B pumilus* strains were subjected to molecular biology–based bacterial identification. The 16S rDNA amplification and sequencing have also identified the 18 COPS, 35A COPS, 35R COPS, 38 COPS, and 40A COPS strains as *B. pumilus*. The 16S rDNA sequence have been subjected to Genbank and the accession numbers are MF692772; MF692773; MF692774; MF692775, and MF692776, respectively. With max scores between 1982 and 2617, *e*-values of 0 and identification scores of 99%, the phylogenetic analysis of 16S rDNA sequences (**Figure 2B**) shows that these five strains are affiliated and very close to each other, except for the 35R COPS strain which is grouped alone. Furthermore, the 4 strains 18 COPS, 35A COPS, 38 COPS, and 40A COPS seem to form one distinct group, and appear affiliated with the reference strain *B. pumilus* ATCC7061. Interestingly this group clustered on the same branch from which the 35R COPS strain derived, this strain forming an isolated ramification. The identification score of 96% for 35R COPS strain suggests that it could be new strain that is not in the NCBI database. This finding prompted us to characterize the secondary metabolites produced by this isolate.

### Characterization of Antibacterial Activities

**FIGURE 3:**
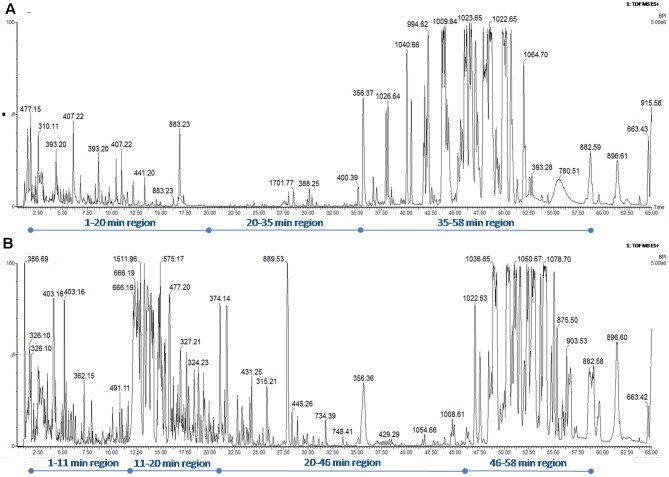
Base-peak intensity-chromatogram (BPI) of CFS issued from the *B. subtilis* 23 COPS **(A)** and *B. pumilus* 35R **(B)** strains. The BPI-chromatograms are normalized to 5 × 10^6^ ions for a better BPI-chromatogram comparison. Elution of the C18-retained molecules were performed using a linear ACN gradient of around 1.3%/min (25–95% ACN in 55 min).

The medium culture (blank data not shown), the CFS issued from the *B. pumilus* 35R and *B. subtilis* 23 COPS strains were subjected to a reverse-phase HPLC-MS/MS analysis on an Acquity-Synapt G2-Si device (Waters). The C18-retained molecules were eluted using a linear ACN gradient of around 1.3%/min (25–95% ACN in 55 min). The BPI-chromatograms (normalized to 5 × 10^6^ ions for a better BPI-chromatogram comparison) are depicted in **Figures 3A,B**, respectively. As expected, the resolution of the C18 core-shell column revealed the presence of many compounds as demonstrated by the number of peaks. The BPI-chromatogram from *B. subtilis* 23 COPS CFS can be divided into 3 zones: (i) the 1–20 min region corresponds to less hydrophobic compounds and shows only a few peaks, (ii) the 20–35 min region where no mass signals are detected and (iii) the 35–58 min regions which corresponds to the elution of lipopeptides (Kalinovskaya et al., 2002) and during which around seven mass signals corresponding to surfactin isoforms are detected (see Supplementary Table S1 for details). The BPI-chromatogram from *B. pumilus* (35R COPS) CFS can be divided into four zones: (i) the 1–11 min region which is similar in terms of mass signals to the same region issued from the 23 COPS CFS BPI-chromatogram, (ii) the 11–20 min region characterized by a very high intensity (5 × 10^15^) of mass signals and which is not present in the 23 COPS CFS BPI-chromatogram, (iii) the 20–46 min region where only a few mass signals are detected and (iv) the 46–58 min region, which also corresponds to the elution zone of lipopeptides, demonstrates that the heavy surfactin isoforms (Supplementary Table S1) produced by the 35R COPS strain are more abundant than in the 23 COPS CFS. Therefore, through this differential approach combining antagonism activity and the HPLC-MS/MS analysis of CFS, we conclude that the antagonism activity of the CFS of *B. pumilus* 35R COPS is mandatory due to compounds in the 11–20 min region of the BPI-chromatogram.

**FIGURE 4:**
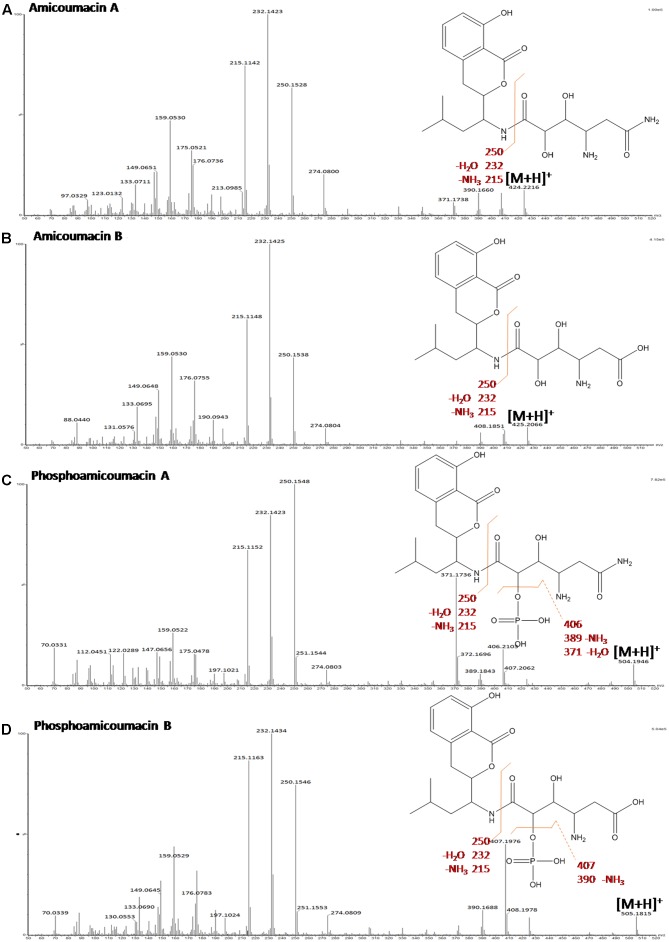
Mass fragmentation patterns obtained by CID of the main abundant forms of amicoumacins: **(A)** amicoumacin A, **(B)** amicoumacin B, **(C)** phosphoamicoumacin A, and **(D)** phosphoamicoumacin B of *B. pumilus* 35R COPS CSF. The spectra were produced using a SYNAPT-G2-Si mass spectrometer (Waters) operating in data-dependent mode.

**Table 2** gathers the main compounds detected by UV-absorbance and mass spectrometry from the 11–20 min region of the BPI-chromatogram. Molecular structures were deduced based on specific UV-absorbance profiles and mass fragmentation patterns (**Figure 4**) obtained by CID. According to the UV-absorbance profile, phosphoamicoumacin B (m/z = 505.2065; RT = 13.19 min) and A (m/z = 504.2140; RT = 12.86 min) are the most abundant molecular forms. Concomitantly, amicoumacin B (m/z = 425.2271; RT = 14.1 min) and A (m/z = 424.2271; RT = 13.42 min) are also detected in mass spectrometry but their UV-signal calculated abundance is lower.

**Table 2:** List of amicoumacins characterized by UV-absorbance and mass spectrometry from the 11–20 min region of the BPI-chromatogram issued from the CFS of *B. pumilus* 35R COPS.

Name	m/z^a^	Mass (Da)	Molecular formula	Retention time (min)	UV λ max (nm)
Amicoumacin A	424.2271	423.2006	C_20_H_29_N_3_O_7_	13.42	247; 313
Amicoumacin B	425.2271	424.1846	C_20_H_28_N_2_O_8_	14.17	247; 313
Phosphoamicoumacin A	504.2140	503.1669	C_20_H_30_N_3_O_10_P	12.86	247; 313
Phosphoamicoumacin B	505.2065	504.1509	C_20_H_29_N_2_O_11_P	13.19	247; 313


*^a^Mass over charge ration (m/z).*

Molecular structure elucidation was performed using the CID-fragmentation patterns shown in **Figures 4A–D**. As illustrated, the fragmentation pattern below the m/z 274.08 is strictly identical from one molecule to another and, combined with the UV-profile common to all compounds, this indicates that all molecules have the same chromophore (3, 4-dihydro-8-hydroxyisocoumarin). The phosphorylated amicoumacins are easily identified by the 98 Da-mass difference, specific to the loss of a phosphoric acid group (H_2_PO_4_). Finally, for each compound, the ion fragments detected in the higher m/z values testify to the successive loss, from the pseudo molecular ions, of NH_3_ and H_2_O groups and therefore demonstrate the presence of an amide group (amicoumacin A forms) and a carboxylic group (amicoumacin B forms).

## Discussion

In this study, using mesophilic culture conditions, 175 bacterial strains were isolated from strains colonizing *A. tonsa* eggs, among which large majority (70%) of these isolates were GNB and a quarter were catalase positive. MALDI-TOF-MS analysis allowed rapid and reliable identification of most of these isolates, at least at the genus level and often at the species level. *In fine* although the real numerical relevance can be different compared to a 16S rDNA sequencing that can be directly performed on copepod eggs, a great diversity of bacterial genera was found, including *Staphylococcus*, *Kocuria*, and *Pseudomonas* strains. Although the copepod-egg isolated strains may have as origin the environment (e.g., adult copepods itself: from mating, egg release, fecal pellets; microalgae), some identified strains are known to be opportunistic pathogens for fishes, humans or both such as *V. anguillarum*, *E. coli*, or *B. cereus*. Bacteria that are harmless to fish but pathogenic to humans (*Listeria, Staphylococcus*, and *Vibrio*) constitute a threat that is all the more important as fish living in polluted waters can carry *Streptococci* and fecal coliforms (Gatesoupe and Lésel, 1998). Previously, Hansen and Bech (1996) reported that the microflora of fecal pellets from another copepod species of Northern hemisphere, *A. tonsa* (Dana) were dominated by *Bacillus*, *Cytophaga/Flavobacterium*, *Vibrio*, and *Pseudomonas*. These bacteria were mainly found attached to the exoskeleton of copepods and to a lesser extent in the gut as well as internally in skeletal muscle tissue. Once more, we illustrate that these strains are generally associated to the *A. tonsa* culture, and this from the laying of eggs. Hence, the copepod eggs could be an elegant way for transporting strains of interest. However, a recent study, based on a dual analysis (denaturing gradient gel electrophoresis (DGGE) and pyrosequencing) targeting the bacterial 16S rRNA gene, unveiled that the microbiota of copepods is dominated by Alphaproteobacteria and Gammaproteobacteria and showed the presence, in lower abundance of *Actinobacteria*, *Verrucomicrobia*, *Firmicutes*, and *Bacteroidetes* (Skovgaard et al., 2015). The identification of the exact pathway of colonization can be developed in future studies by using appropriate experimental protocol. In fact, we did not find in the literature any study focusing on the pathway of bacteria colonization in copepod eggs. But, some recent studies focusing on late developmental stages of copepods (mainly adults) showed that the colonization pathway could be complicated (Dumontet et al., 1996; Rawlings et al., 2007; Almada and Tarrant, 2016). Moreover, some experimental studies confirmed that some bacterial strains (such as *E. coli.*) cannot colonize copepods (Dumontet et al., 1996). Anyway, the pathway of bacterial colonization of copepod eggs was not the central element of our study.

Here, we focused our attention primarily on five strains belonging to the *B. pumilus* species (18 COPS, 35A COPS, 35R COPS, 38 COPS, and 40A COPS), initially identified by MALDI-TOF-MS and then confirmed by sequence analysis of the 16S rDNA, for several reasons. First of all *B. pumilus* had already been described as a potential probiotic bacterium (Duc et al., 2004). Such strains could be easily used in aquaculture, as *Bacillus* species are known for their ability to adapt easily to diverse habitats (Parvathi et al., 2009). Moreover several species of *Bacillus* including *B. cereus*, *B. subtilis*, and *B. pumilus* inhabit coastal and marine environments (Parvathi et al., 2009). Finally, *B. pumilus* has also been reported to be the second most predominant *Bacillus* species in space crafts (Nicholson et al., 2000).

The antagonism capability of the five strains of *B. pumilus* isolated in this study was suggested by their antimicrobial activity against several pathogens (*V. anguillarum, V. alginolyticus, L. innocua, L. monocytogenes*, and *S. aureus*). Interestingly, the probiotic propensity of *B. pumilus* strains have been already reported. A recent study showed that *B. pumilus* H2 isolated from marine sediment, used as probiotic for juvenile shrimp, has strong potential for the prevention or treatment of fish vibriosis in the aquaculture industry (Gao et al., 2017). Previously, Leyton et al. (2012) have shown that *B. pumilus*, issued from a marine strain of the collection of the Laboratorio de Ecología Microbiana of the Universidad de Antofagasta, produces metabolites that significantly inhibit the growth of the pathogen *V. parahaemolyticus*. Another example, a *B. pumilus* strain, isolated from the mid-gut of healthy black tiger shrimp, weakly inhibits the growth of *V. parahaemolyticus* in cross-streaking assays on solid medium but strongly inhibits those of the marine bacterial pathogens *V. alginolyticus*, *V. mimicus*, and *V. harveyi*, and was thought-out commercial potential as a probiotic (Hill et al., 2009).

Through this study, we illustrate that the CFS collected from the culture of our five *B. pumilus* strains inhibit the growth of various *Vibrio* strains. These bacteria are responsible for fish vibriosis which causes lethargy, degeneration of the gills, lymphoid organ, digestive system and epitheliums leading to eventual or rapid mortality (Rasheed, 1989; Deane et al., 2001; Akaylı and Timur, 2002; Haenen et al., 2014). To limit the dissemination of resistant bacteria, the use of antibiotics to fight bacterial diseases in aquaculture farms is now banished or strictly regulated in developed countries (Carbone and Faggio, 2016). Therefore, the use of antagonistic bacteria to limit the development of vibriosis appears as a promising strategy (Carbone and Faggio, 2016), and *Bacillus* seems to be a good candidate for this purpose. Bacteria belonging to this genus display several advantages including an environmental ubiquity, a persistence and stability in the harsh conditions of the gastrointestinal ecosystem since *Bacillus* spores can tolerate such environment and are able to germinate and proliferate within the intestine (Tam et al., 2006). Moreover Prieto et al. (2014) have recently recommended that probiotics should be isolated from the gastrointestinal tract to give them the best chance of surviving in and colonizing the intestine.

The mode of action of probiotics in *in vivo* conditions remains unclear with a number of potentially synergistic or complementary mechanisms proposed, including immunostimulation (Díaz-Rosales et al., 2006; Scharek et al., 2007), inhibition or competition with potential pathogens (Vine et al., 2004; Balcázar and Rojas-Luna, 2007; Decamp et al., 2008) or simply maintenance of healthy and diverse intestinal microbiota. In our case the five isolated strains of *B. pumilus* display antagonism which could be associated with the production of two major families of antimicrobial compounds, the fengycins and the surfactins (Lebbadi et al., 1994; Xu et al., 2014; Mora et al., 2015). The presence of the genes coding for these two lipopeptides was established and could be considered as one of the main mechanisms implicated in the antagonistic effects on the pathogens tested. HPLC-MS/MS analyses performed on the most active CFS from by *B. pumilus* and the inactive CFS from *B. subtilis* isolated in this study reveals that no molecule of the fengycin family is produced by both strains. Concomitantly, the population of surfactin molecules is not so different between each strain and, as demonstrated by HPLC-MS/MS data, only *B. pumilus* produces heavier surfactin isoforms. Similarly, Berrue et al. (2009) have isolated a *B. pumilus* strain (SP21) from a sediment sample collected in the Bahamas and reported its antagonism against *S. aureus* ATCC 10832, *Pseudomonas aeruginosa* ATCC 14210 and *Candida albicans* ATCC 14053. As characterized by spectroscopic methods including 2D-NMR and MS, this strain SP21 produces 5 surfactin analogs, the glycocholic acid, the amicoumacins A and B in addition to 3 lipoamides A–C.

Some strains of the *Bacillus* genus produce natural compounds displaying antagonistic activities against many bacterial, fungal pathogens and are often used as agents for the treatment and/or prevention of different plant and animal infections (Ongena and Jacques, 2008; Lee and Kim, 2011). Their antimicrobial activities are mainly attributed to the production of antibiotic peptide derivatives of the lipopeptide family (Berrue et al., 2009). *B. pumilus* strains have been reported as producing another antimicrobial peptides called pumilucidins which are active against a wide range of microorganisms (Naruse et al., 1990). In this study, we reported that two major families of antimicrobial compounds are produced by the *B. pumilus* strain named 35R COPS associated to copepod eggs: the surfactin and the amicoumacin families.

The amicoumacins A, B, and C are a family of structurally diverse products that possess a broad range of pharmacological properties such as antibacterial, anti-inflammatory, antiulcer, gastroprotective, and anti-*Helicobacter pylori* (Itoh et al., 1981; Hashimoto et al., 2007). A recent study has shown that *B. pumilus* H2 isolated from marine sediment produces an active molecule against *Vibrio* species (Gao et al., 2017). This active molecule was identified as the amicoumacin A, indicating that this *B. pumilus* strain has a strong potential application in the prevention or control of fish vibriosis. In 2007, the MU313B *B. pumilus* strain isolated from a soil sample collected in Japan was shown to produce amicoumacins A, B and their 8′-phospho derivatives. These molecules have been reported to play a crucial role in the anti-*S. aureus* and anti-MRSA activities (Hashimoto et al., 2007). The potential probiotic traits of the *B. pumilus* 35R COPS strain associated to copepod eggs should be investigated further and confirmed by *in vivo* study to establish their efficacy in live animals.

## Conclusion

Copepods are a natural, economical, equilibrated and safe food source for the growth of fish larvae in larviculture. Moreover, through their microbiote (acquired or innate), copepods appear as a natural way of probiotics administration for the fish larvae. Here, from copepod eggs (isolation site) and using the modern, fast, accurate MALDI-TOF-profiling approach, we reported the bacterial identification of 102 (58%) over 175 isolates obtained according to mesophilic culture conditions. Thirty-four strains belong to *Vibrio* genus, 18 to *Staphylococcus* genus, *14* to *Pseudomonas* genus, 25 to *Bacillus* genus. Among the latter, *B. pumilus* and *B. subtilis* strains were subjected to two antagonism tests. The CFS of *B. pumilus* displayed an antagonistic activity against certain species of the *Vibrio*, *Kocuria*, *Listeria*, and *Staphylococcus* genera in contrast to those of *B. subtilis*. These differences in antimicrobial activities have been used for a rapid discrimination of the compounds responsible for the antagonism. Therefore, their CFS compounds were resolved and compared through a differential HPLC-ESI-MS/MS approach. Using this approach, the surfactin family molecules found in each CFS have been rapidly excluded as candidate and compounds found exclusively in the *B. pumilus* CFS, eluted in the 11–20 min region, were characterized using their specific UV-absorbance profiles and their MS/MS fragmentation patterns. Hence, the antagonism activity of *B. pumilus* CFS was indirectly shown to be due to members of the amicoumacin family: the amicoumacin A, the amicoumacin B, the phosphoamicoumacin A and the phosphoamicoumacin B. The potential probiotic propensity of the 35R COPS strain stays still clearly to establish in the future initially through the determination of its *in vitro* survival in gastric and intestinal environments, the determination of hydrophobicity percentage but also other added values and the search of the genes coding for the members of the amicoumacin family.

## Author Contributions

CF and SS co-designed the study and participated in its initiation and coordination, together with TG, BC and DD. MZ, MC, and YB carried out the bacteria culture, the molecular biology experiments and the antagonism research. Chromatography and mass spectrometry experiments were performed by MC and MZ. All authors participated in the result interpretations and helped draft the manuscript. All authors have read and approved the final manuscript.

## Conflict of Interest Statement

The authors declare that the research was conducted in the absence of any commercial or financial relationships that could be construed as a potential conflict of interest.

## Abbreviations

ACN: acetonitrile
BLAST: basic local alignment search tool
BPI: base peak intensity
BTS: bacterial test standard
CFS: cell-free supernatants
CID: collision-induced dissociation
COPS: strain acronym for copepods
ESI: electrospray ionization
GNB: Gram-negative bacteria
GPB: Gram-positive bacteria
HCCA: α-cyano-4-hydroxycinnamic acid
LOG: Laboratory Oceanography and Geosciences
MALDI-TOF-MS: matrix-assisted laser desorption/ionization-time of flight-mass spectrometry
MSP: main spectral projection
NCBI: National Center for Biotechnology Information
PCR: polymerase chain reaction
SSB: sterile saline buffer
TFA: trifluoroacetic acid

## References

[B1] Abate T. G. Nielsen R. Nielsen M. Drillet G. Jepsen P. M. Hansen B. W. (2015). Economic feasibility of copepod production for commercial use: result from a prototype production facility. *Aquaculture* 436 72–79. 10.1016/j.aquaculture.2014.10.012

[B2] Akaylı T. Timur G. (2002). Vibriosis in gilthead sea bream (*Sparus aurata* L.) in farms in the aegean sea coast of Turkey. *Turk. J Fish. Aquat. Sci.* 2 89–91.

[B3] Al Atya A. K. Drider-Hadiouche K. Ravallec R. Silvain A. Vachee A. Drider D. (2015). Probiotic potential of *Enterococcus faecalis* strains isolated from Meconium. *Front. Microbiol.* 6:227 10.3389/fmicb.2015.00227 PMC4382979 25883590

[B4] Almada A. A. Tarrant A. M. (2016). Vibrio elicits targeted transcriptional responses from copepod hosts. *FEMS Microbiol. Ecol.* 92 1–11. 10.1093/femsec/fiw072 27056917

[B5] Aly S. M. Abdel-Galil Ahmed Y. Abdel-Aziz Ghareeb A. Mohamed M. F. (2008). Studies on *Bacillus subtilis* and *Lactobacillus acidophilus*, as potential probiotics, on the immune response and resistance of *Tilapia nilotica* (*Oreochromis niloticus*) to challenge infections. *Fish Shellfish Immunol.* 25 128–136. 10.1016/j.fsi.2008.03.013 18450477

[B6] Austin B. Austin D. A. (1993). “Control,” in *Bacterial Fish Pathogens : Disease of Farmed and Wild Fish*, ed. Schuster S. (Chichester: Springer), 693–706.

[B7] Balcázar J. L. Rojas-Luna T. (2007). Inhibitory activity of probiotic *Bacillus subtilis* UTM 126 against Vibrio species confers protection against Vibriosis in Juvenile Shrimp (*Litopenaeus vannamei*). *Curr. Microbiol.* 55 409–412. 10.1007/s00284-007-9000-0 17680306

[B8] Berrue F. Ibrahim A. Boland P. Kerr R. G. (2009). Newly isolated marine *Bacillus pumilus* (SP21): a source of novel lipoamides and other antimicrobial agents. *Pure Appl. Chem.* 81 1027–1031. 10.1351/PAC-CON-08-09-25

[B9] Carbone D. Faggio C. (2016). Importance of prebiotics in aquaculture as immunostimulants. Effects on immune system of *Sparus aurata* and *Dicentrarchus labrax*. *Fish Shellfish Immunol.* 54 172–178. 10.1016/j.fsi.2016.04.011 27074444

[B10] Colwell R. R. Huq A. (1998). Global microbial ecology: biogeography and diversity of vibrios as a model. *J. Appl. Microbiol.* 85 134S–137S. 10.1111/j.1365-2672.1998.tb05292.x 21182702

[B11] Deane E. E. Li J. Woo N. Y. (2001). Hormonal status and phagocytic activity in sea bream infected with vibriosis. *Comp. Biochem. Physiol. Part B* 129 687–693. 10.1016/S1096-4959(01)00369-4 11399506

[B12] Decamp O. Moriarty D. J. W. Lavens P. (2008). Probiotics for shrimp larviculture: review of field data from Asia and Latin America. *Aquac. Res.* 39 334–338. 10.1111/j.1365-2109.2007.01664.x

[B13] Díaz-Rosales P. Salinas I. Rodríguez A. Cuesta A. Chabrillón M. Balebona M. C. (2006). Gilthead seabream (*Sparus aurata* L.) innate immune response after dietary administration of heat-inactivated potential probiotics. *Fish Shellfish Immunol.* 20 482–492. 10.1016/j.fsi.2005.06.007 16169250

[B14] Drago L. Mattina R. Nicola L. Rodighiero V. De Vecchi E. (2011). Macrolide resistance and in vitro selection of resistance to antibiotics in lactobacillus isolates. *J. Microbiol.* 49 651–656. 10.1007/s12275-011-0470-1 21887650

[B15] Drillet G. Jørgensen N. O. G. Sørensen T. F. Ramløv H. Hansen B. W. (2006). Biochemical and technical observations supporting the use of copepods as live feed organisms in marine larviculture. *Aquac. Res.* 37 756–772. 10.1111/j.1365-2109.2006.01489.x

[B16] Duc L. H. Hong H. A. Barbosa T. M. Henriques A. O. Cutting S. M. (2004). Characterization of Bacillus probiotics available for human use. *Appl. Environ. Microbiol.* 70 2161–2171. 10.1128/AEM.70.4.2161-2171.2004 15066809 PMC383048

[B17] Dumontet S. Krovacek K. Baloda S. B. Grottoli R. Pasquale V. Vanucci S. (1996). Ecological relationship between aeromonas and vibrio spp. and planktonic copepods in the coastal marine environment in Southern Italy. *Comp. Immunol. Microbiol. Infect. Dis.* 19 245–254. 10.1016/0147-9571(96)00012-4 8800550

[B18] Franco S. C. Augustin C. B. Geffen A. J. Teresa Dinis M. (2017). Growth, egg production and hatching success of *Acartia tonsa* cultured at high densities. *Aquaculture* 468 569–578. 10.1016/j.aquaculture.2016.10.044

[B19] Gao X. Y. Liu Y. Miao L. L. Li E. W. Hou T. T. Liu Z. P. (2017). Mechanism of anti - vibrio activity of marine probiotic strain *Bacillus pumilus* H2, and characterization of the active substance. *AMB Express* 7:23 10.1186/s13568-017-0323-3 PMC5241254 28097594

[B20] Gatesoupe F. J. Lésel R. (1998). Flore digestive des poissons : approche environnementale. *Cah. Etud. Rech. Francophones Agric.* 7 29–35.

[B21] González M. T. Castro R. Muñoz G. López Z. (2016). Sea lice (Siphonostomatoida: Caligidae) diversity on littoral fishes from the south-eastern pacific coast determined from morphology and molecular analysis, with description of a new species (Lepeophtheirus Confusum). *Parasitol. Int.* 65 685–695. 10.1016/j.parint.2016.08.006 27580816

[B22] Gugliandolo C. Irrera G. P. Lentini V. Maugeri T. L. (2008). Pathogenic Vibrio, Aeromonas and *Arcobacter* Spp. Associated with Copepods in the Straits of Messina (Italy). *Mar. Pollut. Bull.* 56 600–606. 10.1016/j.marpolbul.2007.12.001 18215401

[B23] Haenen O. L. M. Fouz B. Amaro C. Isern M. M. Mikkelsen H. Zrncic S. (2014). Vibriosos in aquaculture. *Eur. Assoc. Fish Pathol. Bull.* 34 138–148.

[B24] Hagemann A. Øie G. Evjemo J. O. Olsen Y. (2016). Effects of light and short-term temperature elevation on the 48-H hatching success of cold-stored *Acartia tonsa* dana eggs. *Aquac. Int.* 24 57–68. 10.1007/s10499-015-9908-5

[B25] Hall B. G. (2013). Building phylogenetic trees from molecular data with MEGA. *Mol. Biol. Evol.* 30 1229–1235. 10.1093/molbev/mst012 23486614

[B26] Hansen B. Bech G. (1996). Bacteria associated with a marine planktonic copepod in culture. I. Bacterial genera in seawater, body surface, intestines and fecal pellets and succession during fecal pellet degradation. *J. Plankton Res.* 18 257–273. 10.1093/plankt/18.2.257

[B27] Hansen B. W. Buttino I. Cunha M. E. Drillet G. (2016). Embryonic cold storage capability from seven strains of Acartia Spp. Isolated in Different Geographical Areas. *Aquaculture* 457 131–139. 10.1016/j.aquaculture.2016.02.024

[B28] Hashimoto M. Taguchi T. Nishida S. Ueno K. Koizumi K. Aburada M. (2007). Isolation of 8 -Phosphate ester derivatives of amicoumacins : structure-activity relationship of hydroxy amino acid moiety. *Matrix* 60 752–756. 10.1038/ja.2007.99 18277000

[B29] Hill J. E. Baiano J. C. F. Barnes A. C. (2009). Isolation of a novel strain of *Bacillus pumilus* from Penaeid shrimp that is inhibitory against marine pathogens. *J. Fish Dis.* 32 1007–1016. 10.1111/j.1365-2761.2009.01084.x 19573134

[B30] Højgaard J. K. Jepsen P. M. Hansen B. W. (2008). Salinity-induced quiescence in eggs of the calanoid copepod *Acartia tonsa* (dana): a simple method for egg storage. *Aquac. Res.* 39 828–836. 10.1111/j.1365-2109.2008.01936.x

[B31] Hong H. A. Duc le H. Cutting S. M. (2005). The use of bacterial spore formers as probiotics. *FEMS Microbiol. Rev.* 29 813–835. 10.1016/j.femsre.2004.12.001 16102604

[B32] Humes A. G. (1994). *How Many Copepods? In Ecology and Morphology of Copepods.* Dordrecht: Springer, 1–7. 10.1007/978-94-017-1347-4_1

[B33] Itoh J. Shomura T. Omoto S. Miyado S. Yuda Y. Shibata U. (1981). Isolation, physicochemical properties and biological activities of amicoumacins produced by *Bacillus pumilus*. *Agric. Biol. Chem.* 46 1255–1259.

[B34] Kalinovskaya N. I. Kuznetsova T. A. Ivanova E. P. Romanenko L. A. Voinov V. G. Huth F. (2002). Characterization of surfactin-like cyclic depsipeptides synthesized by *Bacillus pumilus* from Ascidian *Halocynthia aurantium*. *Mar. Biotechnol.* 4 179–188. 10.1007/s10126-001-0084-4 14961278

[B35] Lebbadi M. Gálvez A. Maqueda M. Martínez-Bueno M. Valdivia E. (1994). Fungicin M4: a narrow spectrum peptide antibiotic from Bacillus licheniformis M-4. *J. Appl. Bacteriol.* 77 49–53. 10.1111/j.1365-2672.1994.tb03043.x 7928782

[B36] Lee H. Kim H.-Y. (2011). Lantibiotics, class I bacteriocins from the Genus Bacillus. *J. Microbiol. Biotechnol.* 21 229–235. 21464591

[B37] Leyton Y. Borquez J. Darias J. Cueto M. Díaz-Marrero A. R. Riquelme C. (2012). Diketopiperazines produced by an Bacillus species inhibits *Vibrio parahaemolyticus*. *J. Aquac. Res. Dev.* 3 144.

[B38] Mauchline J. (1998). *The Biology of Calanoid Copepods.* Cambridge, MA: Academic Press.

[B39] Mora I. Cabrefiga J. Montesinos E. (2015). Cyclic lipopeptide biosynthetic genes and products, and inhibitory activity of plant-associated Bacillus against phytopathogenic bacteria ed. Vittorio Venturi. *PLOS ONE* 10:e0127738 10.1371/journal.pone.0127738 PMC4449161 26024374

[B40] Moriarty D. J. W. (1998). Control of luminous Vibrio Species in penaeid aquaculture ponds. *Aquaculture* 164 351–358. 10.1016/S0044-8486(98)00199-9

[B41] Nacef M. Chevalier M. Chollet S. Drider D. Flahaut C. (2016). MALDI-TOF mass spectrometry for the identification of lactic acid bacteria isolated from a French cheese: the maroilles. *Int. J. Food Microbiol.* 247 2–8. 10.1016/j.ijfoodmicro.2016.07.005 27423415

[B42] Naruse N. Tenmyo O. Kobaru S. Kamei H. Miyaki T. Konishi M. (1990). Pumilacidin, a complex of new antiviral antibiotics. production, isolation, chemical properties, structure and biological activity. *J. Antibiotics* 43 267–280. 10.7164/antibiotics.43.267 2157695

[B43] Nicholson W. L. Munakata N. Horneck G. Melosh H. J. Setlow P. (2000). Resistance of Bacillus endospores to extreme terrestrial and extraterrestrial environments. *Microbiol. Mol. Biol. Rev.* 64 548–572. 10.1128/MMBR.64.3.548-572.2000 10974126 PMC99004

[B44] Ongena M. Jacques P. (2008). Bacillus lipopeptides: versatile weapons for plant disease biocontrol. *Trends Microbiol.* 16 115–125. 10.1016/j.tim.2007.12.009 18289856

[B45] Pan Y.-J. Souissi A. Hwang J.-S. Souissi S. (2015). Short Communication artificially cold-induced quiescent egg viability of the tropical copepod *Acartia bilobata* (Copepoda, Calanoida). *Aquac. Res.* 2015 1–6.

[B46] Parvathi A. Krishna K. Jose J. Nair S. (2009). Biochemical and molecular characterization of *Bacillus pumilus* isolated from coastal environment in Cochin, India. *Braz. J. Microbiol.* 40 269–275. 10.1590/S1517-838220090002000012 24031357 PMC3769717

[B47] Prieto M. L. O’Sullivan L. Tan S. P. McLoughlin P. Hughes H. Gutierrez M. (2014). In vitro assessment of marine Bacillus for use as livestock probiotics. *Mar. Drugs* 12 2422–2445. 10.3390/md12052422 24796302 PMC4052298

[B48] Rasheed V. (1989). Vibriosis outbreak among cultured seabream (*Acanthopagrus cuvieri*) broodstock in Kuwait. *Aquaculture* 76 189–197. 10.1016/0044-8486(89)90072-0

[B49] Rawlings T. K. Ruiz G. M. Colwell R. R. (2007). Association of *Vibrio cholerae* O1 El Tor and O139 Bengal with the copepods *Acartia tonsa* and *Eurytemora affinis*. *Appl. Environ. Microbiol.* 73 7926–7933. 10.1128/AEM.01238-07 17951440 PMC2168156

[B50] Roddie B. D. Leakey R. J. G. Berry A. J. (1984). Salinity-temperature tolerance and osmoregulation in *Eurytemora affinis* (Poppe) (Copepoda : Calanoida) in relation to its distribution in the zooplankton of the upper reaches of the forth estuary. *J. Exp. Mar. Biol. Ecol.* 79 191–211. 10.1016/0022-0981(84)90219-3

[B51] Scharek L. Altherr B. J. Tölke C. Schmidt M. F. G. (2007). Influence of the probiotic *Bacillus cereus* Var. Toyoi on the Intestinal Immunity of Piglets. *Vet. Immunol. Immunopathol.* 120 136–147. 10.1016/j.vetimm.2007.07.015 17870185 PMC7112577

[B52] Skovgaard A. Castro-Mejia J. L. Hansen L. Nielsen D. S. (2015). Host-specific and pH-dependent microbiomes of copepods in an extensive rearing system ed. Jiang-Shiou Hwang. *PLOS ONE* 10:e0132516 10.1371/journal.pone.0132516 PMC4500450 26167852

[B53] Souissi A. Souissi S. Hansen B. W. (2016). Physiological improvement in the copepod *Eurytemora affinis* through thermal and multi-generational selection. *Aquac. Res.* 47 2227–2242. 10.1111/are.12675

[B54] Stancheva S. Souissi A. Ibrahim A. Barras A. Spriet C. Souissi S. (2015). Lipid nanocapsules as a new delivery system in copepods: toxicity studies and optical imaging. *Colloids Surf. B* 135 441–447. 10.1016/j.colsurfb.2015.07.082 26280818

[B55] Sun Y. Z. Yang H. L. Huang K. P. Ye J. D. Zhang C. X. (2013). Application of autochthonous Bacillus bioencapsulated in Copepod to grouper *Epinephelus coioides* Larvae. *Aquaculture* 392–395, 44–50. 10.1016/j.aquaculture.2013.01.037

[B56] Tam N. K. M. Uyen N. Q. Hong H. A. Duc L. H. Hoa T. T. Serra C. R. (2006). The intestinal life cycle of *Bacillus subtilis* and close relatives. *J. Bacteriol.* 188 2692–2700. 10.1128/JB.188.7.2692-2700.2006 16547057 PMC1428398

[B57] Tlili S. Ovaert J. Souissi A. Ouddane B. Souissi S. (2016). Acute toxicity, uptake and accumulation kinetics of nickel in an invasive copepod species: *Pseudodiaptomus marinus*. *Chemosphere* 144 1729–1737. 10.1016/j.chemosphere.2015.10.057 26519805

[B58] Vine N. G. Leukes W. D. Kaiser H. Daya S. Baxter J. Hecht T. (2004). Competition for attachment of aquaculture candidate probiotic and pathogenic bacteria on fish intestinal mucus. *J. Fish Dis.* 27 319–326. 10.1111/j.1365-2761.2004.00542.x 15189372

[B59] Xu H. M. Rong Y. J. Zhao M. X. Song B. Chi Z. M. (2014). Antibacterial activity of the lipopetides produced by *Bacillus amyloliquefaciens* M1 against multidrug-resistant Vibrio Spp. Isolated from diseased marine animals. *Appl. Microbiol. Biotechnol.* 98 127–136. 10.1007/s00253-013-5291-1 24132666

