# Supplementary material for: Isolation and Characterization of Bacteria Colonizing Acartia tonsa Copepod Eggs and Displaying Antagonist Effects against Vibrio anguillarum, Vibrio alginolyticus and Other Pathogenic Strains
Source: Front Microbiol. 2017 Oct 6;8:1919. doi: 10.3389/fmicb.2017.01919 (PMC5649146; doi:10.3389/fmicb.2017.01919)
Supplement: Supplementary file 1 [file Table_1.DOCX]

Supplementary Material

**Isolation and characterization of bacteria from *Acartia tonsa* copepod eggs, displaying antagonist effects against *Vibrio anguilarum*, *Vibrio alginolyticus* and other pathogenic strains.**

**Mahammed ZIDOUR^1^, Mickaël CHEVALIER^1^, Yanath BELGUESMIA^1^, Benoit CUDENNEC^1^, Thierry GRARD^1^, Djamel DRIDER^1^, Sami SOUISSI^2^ and Christophe FLAHAUT^1^***

*** Correspondence:**Dr. Christophe FLAHAUT

christophe.flahaut@univ-artois.fr

# Supplementary Data (Table)

Table S1: List of lipopeptides of the surfactin family found in *B. subtilis* (23 COPS) and *B. pumilus* (35R COPS) strains.

| **Name** | **m/z** | **Mass (Da)** | **Molecular Formula** | ***Bacillus subtilis*** | ***Bacillus pumilus*** |
| --- | --- | --- | --- | --- | --- |
| surfactin C12 | 994.6165 | 993.6362 | C50H87N7O13 | x |  |
| surfactin C13 | 1008.6331 | 1007.6518 | C51H89N7O13 | x | x |
| surfactin C14 | 1022.6504 | 1021.6675 | C52H91N7O13 | x | x |
| (Val)surfactin C15 | 1036.6672 | 1035.6831 | C53H93N7O13 | x | x |
| (Ile)surfactin C15 | 1050.6830 | 1049.6988 | C54H95N7O13 | x | x |
| surfactin C16 | 1064.6965 | 1063.7144 | C55H97N7O13 | x | x |
| surfactin C17 | 1078.7126 | 1077.7301 | C56H99N7O13 | x | x |

**
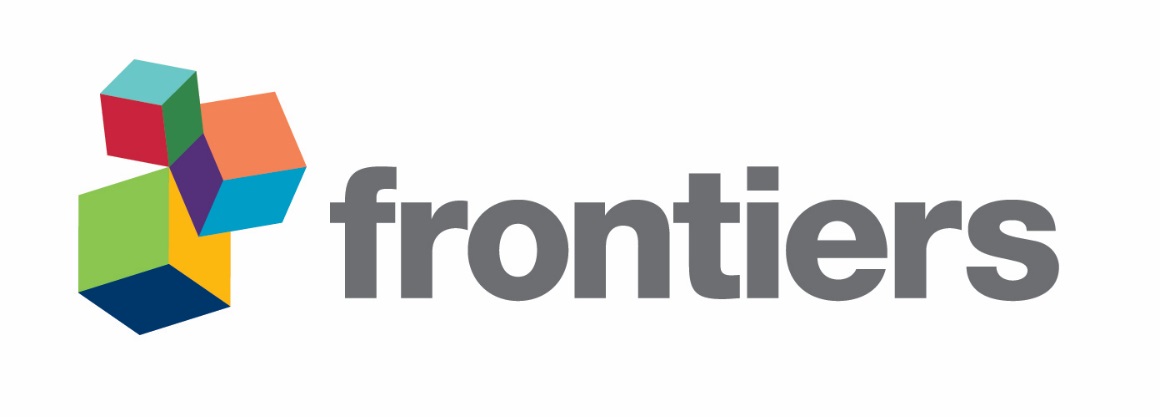
**
